# The Treatment of COVID-19 Purgatory Syndrome With Tocilizumab and Steroids

**DOI:** 10.7759/cureus.22614

**Published:** 2022-02-25

**Authors:** Vijairam Selvaraj, Arkadiy Finn, Jennifer Li, Kwame Dapaah-Afriyie

**Affiliations:** 1 Internal Medicine, Miriam Hospital, Providence, USA; 2 Hospital Medicine, Miriam Hospital, Providence, USA; 3 Internal Medicine, Brown University, Providence, USA

**Keywords:** mis-a, sars-cov2, sars-cov-2, hyperinflammatory syndrome, cps, covid19 purgatory syndrome, chis, hyperinflammation, covid, covid19

## Abstract

Hyperinflammation is a key component of severe coronavirus disease 2019 (COVID-19) and is associated with poor outcomes. It is imperative to distinguish severe COVID-19 from hyperinflammatory syndromes such as multisystem inflammatory syndrome (MIS) and hemophagocytic lymphohistiocytosis. There is a subset of post-COVID-19 patients who present with some symptoms characteristic of MIS in adults (MIS-A) yet do not meet all the criteria for a diagnosis. We describe the unique case of a patient with this kind of presentation who clinically improved following tocilizumab and corticosteroid usage.

## Introduction

Coronavirus disease 2019 (COVID-19) continues to be a global public health crisis, as its clinical spectrum continues to evolve. Yet its clinical and pathophysiological characterization is still not clearly defined. In May 2020, a multisystem inflammatory syndrome in children (MIS-C) associated with COVID-19 was described [1,2]. The illness was characterized by cardiac dysfunction, shock, severely elevated inflammatory markers, and positive severe acute respiratory syndrome coronavirus 2 (SARS-CoV-2) serology. In October 2020, an illness referred to as multisystem inflammatory syndrome in adults (MIS-A) was described. Since then, MIS-A has been increasingly recognized as a late sequela or complication of COVID-19 [3]. However, a few patients do not fully meet MIS-A criteria and only display its symptoms several weeks after their initial infection, and may appear to live in a viral purgatory. We describe the unique case of a patient who tested positive for COVID-19 yet continued to experience persistent fever and gastrointestinal symptoms five weeks later while not meeting the criteria for MIS-A.

## Case presentation

**Figure 1:**
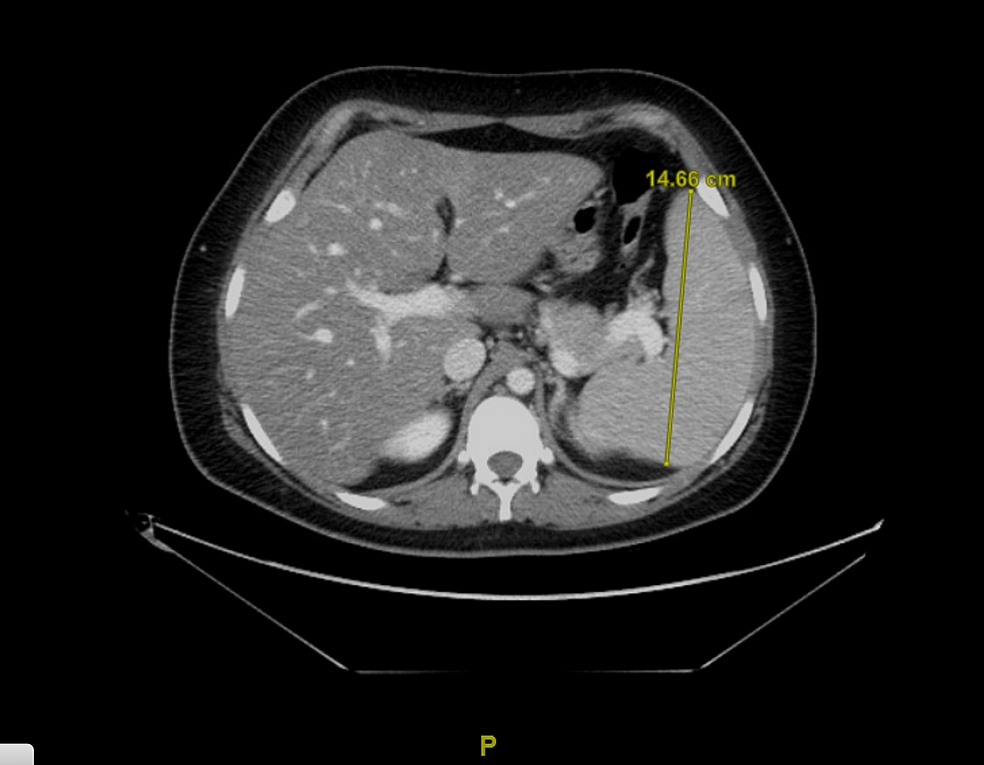
CT abdomen and pelvis with contrast showing splenomegaly measuring 14 cm CT: computed tomography

A 27-year-old female with a history of COVID-19 infection in December 2021 presented to the hospital in February 2022 with recurrent fever and an inability to tolerate oral intake. She had been hospitalized two weeks prior for persistent fevers, vomiting, and generalized malaise. A physical exam at that time had revealed erythematous maculopapular rash over her shins. Her respiratory pathogen panel had been positive for parainfluenza, coxsackievirus 3, and SARS-CoV-2. Imaging had revealed prominent and enlarged retroperitoneal and pelvic lymph nodes and splenomegaly measuring up to 14 cm (Figure 1). With the resolution of her viral illness, her cell counts had improved spontaneously, requiring no further intervention. An inguinal lymph node biopsy had shown likely reactive lymphadenopathy with no evidence of lymphoma. She had been discharged home with ibuprofen and topical ointment for her rash.

She returned to the hospital in February 2022 due to nausea, vomiting, abdominal pain, and persistent fever. She reported a 20-pound weight loss along with arthralgias. At the time of admission, she was noted to have a temperature of 102.1 °F, blood pressure of 100/40 mmHg, and a heart rate of 134 beats/minute. Her physical exam was remarkable for pink-to-red morbilliform plaques scattered diffusely across bilateral upper and lower extremities and trunk. Conjunctivae were clear. There was evidence of synovitis at both wrists, right knee, right ankle joints, and her right forefoot. Table 1presents her labs at the initial and subsequent presentations. In addition, her anti-receptor binding domain (RBD) IgG antibodies were noted to be >25,000, N protein immunoglobulin G (IgG) index was 3.40 (normal level: <1.4), and S protein IgM index was 3.32 (normal level: <1). Antinuclear antibodies (ANA) were 1:40, triglycerides were 376 mg/dl (normal level: <150 mg/dl), and natural killer (NK)-cell activity was 22 (normal range: 8-53.6 LU 10). An extensive workup was otherwise unremarkable. Infectious diseases, rheumatology, dermatology, and hematology were consulted. She was given one dose of tocilizumab (8 mg/kg) for possible COVID-19 hyperinflammatory syndrome (cHIS). She was also started on oral prednisone as per rheumatology recommendations. Her fevers resolved, C-reactive protein (CRP) decreased from 150 to 41 mg/L, and she showed clinical improvement.

**Table 1:** Labs at initial and subsequent presentations CRP: C-reactive protein; AST: aspartate aminotransferase; ALT: alanine transaminase

Labs (reference range)	Initial presentation	Subsequent presentation
Hemoglobin (11-15 g/dl)	9.9 g/dl	10.8 g/dl
Platelets (150,000-400,000)	118,000	193,000
Ferritin (10-120 ng/ml)	>16,500 ng/ml	13,838 ng/ml
CRP (0-10 mg/L)	172.84 mg/dl	149.99 mg/dl
AST (6-45 IU/L)	160 IU/L	78 IU/L
ALT (10-42 IU/L)	58 IU/L	75 IU/L

## Discussion

Scientists have agreed on a biphasic model of COVID-19, with an initial viremic phase followed by a host hyperinflammatory phase in select patients with a dysregulated immune response [4]. The terminology of hyperinflammatory syndromes and disorders has been a subject of much debate. Patients with severe COVID-19 may have hypercytokinemia secondary to macrophage activation and a self-perpetuating cycle of cytokine production [5]. "Cytokine storm" or "cytokine release syndrome" has been used as a blanket term to describe hyperinflammation in COVID-19. This may be misleading as the hypercytokinemia may be less marked in COVID-19 than in other critical hyperinflammatory syndromes, and there may be other alternative mechanisms of hyperinflammation in COVID-19 [6]. The long-term consequences of this disease include long COVID and MIS-A. MIS-A is a late sequela of the hyperinflammatory phase or a delayed inflammatory phase, driven by an overexuberant and unchecked immune response [3]. The Centers for Disease Control and Prevention (CDC) guidelines defining MIS-A were largely adopted from guidelines and criteria used in children (MIS-C).

To diagnose MIS-A as per CDC definition, patients must meet three criteria: at least one of the two primary criteria - severe cardiac illness, or rash and conjunctivitis - plus the presence of neurologic signs and symptoms, hypotension/shock, gastrointestinal symptoms, or thrombocytopenia, as well as laboratory evidence of severe inflammation and SARS-CoV-2 infection [7]. Some patients may only meet a few of the current MIS-A criteria in this spectrum of hyperinflammatory responses. We propose that there exists an "intermediate" group of patients classified as incomplete MIS-A or patients with post-cHIS/COVID-19 purgatory syndrome (CPS). A few studies have looked at different criteria and scoring systems to define cHIS [8-10]. However, this is yet to be fully elucidated. cHIS/CPS is difficult to define and has a heterogenous presentation with overlapping features involving severe COVID-19, MIS-A, and other hyperinflammatory syndromes such as hemophagocytic lymphohistiocytosis (HLH) or adult-onset Still's disease (AOSD). Our patient had a fever, rash, thrombocytopenia, hypotension, gastrointestinal symptoms, elevated inflammatory markers, and evidence of prior SARS-CoV-2 infection. In the absence of severe cardiac illness and conjunctival involvement, the findings were consistent with cHIS/CPS.

The most common differential diagnoses of hyperinflammatory syndromes include HLH, AOSD, and adult Kawasaki disease. The most commonly encountered one is secondary HLH (sHLH), which typically occurs in patients with an underlying process such as infection, malignancy, or an autoimmune disorder. HLH due to a rheumatologic or autoinflammatory disease is also known as macrophage activation syndrome (MAS). To diagnose HLH, patients must meet five of the following eight criteria: fever; splenomegaly; peripheral blood cytopenia involving at least two cell lines; hypertriglyceridemia or hypofibrinogenemia or both; hemophagocytosis in the spleen, bone marrow, lymph nodes, or liver; low or absent NK-cell activity; a ferritin level ≥500 μg/L; and an elevated soluble interleukin-2 (IL-2) receptor α levels [11]. Patients with cHIS/CPS differ from those with sHLH and have distinct characteristics. Patients with cHIS/CPS are more likely to be hypotensive, have significant GI symptoms, thrombocytopenia, and evidence of prior SARS-CoV-2 infection. HLH/MAS, cHIS/CPS, and AOSD share common phenotypes on a spectrum of inflammation. However, they all have distinct treatment options and outcomes. Our patient did not meet five out of eight criteria for HLH (only fever, splenomegaly, elevated ferritin, and hypertriglyceridemia were present). She did not meet Yamaguchi or Fautrel criteria for AOSD either [12].

Cycle threshold (Ct) values are used to measure the number of polymerase chain reaction (PCR) cycles required to amplify the targeted viral nucleic acid to a detectable level and are inversely related to the viral load. Low Ct values indicate a higher viral density, and high Ct values generally indicate a lower viral density. Although Ct has no well-defined cutoff values, it is difficult to detect SARS-CoV-2 when Ct values are ≥30 [13,14]. Our patient’s Ct value at the end of December 2021 was 15. However, at the time of her presentation in February 2022, the Ct value had increased to 43. This implies a lower viral density and a lower likelihood of shedding. Patients infected with COVID-19 usually have both anti-spike and anti-nucleocapsid antibodies. However, anti-nucleocapsid antibodies are typically absent in SARS-CoV-2-naïve, vaccinated individuals [15]. Our patient had significantly elevated anti-RBD IgG antibodies along with nucleocapsid IgG antibodies. Her clinical response to tocilizumab suggests that a post-infectious immune dysregulation phenomenon drove her underlying inflammatory process.

Treatment of this condition largely depends on immunomodulation with glucocorticoids, intravenous immunoglobulin (IVIG), IL-1 inhibitors such as anakinra, or IL-6 inhibitors such as tocilizumab. The cytokine storm involves several cytokines and chemokines such as IL-2, IL-6, and tumor necrosis factor α (TNFα). Among these, IL-6 plays a central role by promoting the proliferation of myeloid precursor cells, synthesis of CRP, and growth and activation of leucocytes. Elevated levels of IL-6 also suppress T-lymphocytes and macrophages, resulting in a decreased ability of the immune system to clear SARS-CoV-2 [16,17]. In our case, the IL-6 level was 18.8 pg/mL (normal value: <2), and tocilizumab was administered following consultation with rheumatology. The exclusion of concurrent bacterial, other viral illnesses, fungal infections, and active tuberculosis is essential. IVIG is the first-line therapy for MIS-A; although our patient did not meet the criteria, it would have been used if she had not responded to tocilizumab [18].

## Conclusions

To our knowledge, this is the first reported case of a proposed cHIS/CPS. This may serve as a reference case for future studies. Existing criteria may need to be revised or broadened to include these "intermediate" groups of patients. Severe COVID-19, cHIS/CPS, and MIS-A exist along the spectrum of inflammation with occasional overlapping features and timelines. Clinicians must have a high index of suspicion as timely and proper recognition of a hyperinflammatory state associated with COVID-19 can be helpful in reducing morbidity and mortality. A multidisciplinary approach may be necessary given the multisystem involvement to exclude other differential diagnoses and to formulate the best treatment plan. Further studies are needed to define this condition better and to understand the underlying pathophysiology to help guide treatment decisions.
